# Effectiveness of an app-based intervention to reduce substance use, gambling, and digital media use in vocational school students: study protocol for a randomized controlled trial

**DOI:** 10.1186/s13063-022-06231-x

**Published:** 2022-04-08

**Authors:** Nicolas Arnaud, Johanna Weymann, Kirsten Lochbühler, Benjamin Pietsch, Monika Rossa, Ludwig Kraus, Rainer Thomasius, Reiner Hanewinkel, Matthis Morgenstern

**Affiliations:** 1 German Centre for Addiction Research in Childhood and Adolescence, University Medical Centre Hamburg-Eppendorf, Hamburg, Germany; 2 IFT Institut für Therapieforschung, Munich, Germany; 3 IFT-Nord Institute for Therapy and Health Research, Kiel, Germany

**Keywords:** Prevention, Vocational students, Voluntary commitment, Abstinence, Substance use, Internet-related problems, Cluster-randomized controlled trial

## Abstract

**Background:**

Substance-related and addictive disorders are among the most common mental disorders in adolescence and young adulthood. Vocational school students are a risk group for problematic substance use and addictive behavior. However, the availability of evidence-based prevention concepts and programs is underdeveloped in the vocational school setting.

**Methods/design:**

A two-arm cluster randomized waitlist-controlled trial will be conducted to evaluate the effectiveness of an app-based intervention to decrease substance use, gambling, and digital media use in vocational school students in Germany. Vocational students will participate in an app-based intervention that is designed to support voluntary commitment to abstain from or reduce substance or digital media use over a period of 2 weeks. The “education-as-usual” control arm will have access to the intervention after data collection is completed. One of the primary outcome measures will be the use of alcohol, nicotine, and digital media 30 days after the intervention. Several secondary outcome measures will also be included, such as cannabis consumption, gambling, symptoms of stress, physical activity, mindfulness, well-being, impulsivity and sensation seeking, and readiness to change. A total of 4500 vocational students from 225 classes will be recruited and randomized across three German federal states.

**Discussion:**

This study protocol describes the design of an RCT testing the effectiveness of an app-based intervention to reduce addictive behaviors in vocational school students. It is expected that this approach will be feasible for and effective in the vocational school setting and that the study provides comprehensive information on the key factors involved in temporary abstaining or reducing substance or digital media use.

**Trial registration:**

German Clinical Trials Register DRKS00023788. Registered on 20 January 2021

## Administrative information

Note: The numbers in curly brackets in this protocol refer to SPIRIT checklist item numbers. The order of the items has been modified to group similar items (see http://www.equator-network.org/reporting-guidelines/spirit-2013-statement-defining-standard-protocol-items-for-clinical-trials/).

**Table:** 

Title {1}	Effectiveness of an app-based intervention to reduce substance use, gambling, and digital media use in vocational school students: study protocol for a randomized controlled trial
Trial registration {2a and 2b}.	Trial registration number DRKS00023788. Registered on 20 January 2021.
Protocol version {3}	The current protocol version is version 1.0, dated from 02 December 2021.
Funding {4}	The trial is funded by the Federal Ministry of Health Germany (BMG, grant number: ZMVI1-2519DSM216). The funding period is from 01 August 2019 to 31 December 2022. The funding source has no role in the design of this study and will not have any role in its execution, analyses, interpretation of the data, or decision to submit results.
Author details {5a}	NA, JW, RT: German Centre for Addiction Research in Childhood and Adolescence at University Medical Centre Hamburg-Eppendorf, Hamburg, Germany; KL, MR, LK: IFT Institut für Therapieforschung München, Germany; MM, BP, RH: Institute for Therapy and Health Research, IFT-Nord.
Name and contact information for the trial sponsor {5b}	Institute for Therapy and Health Research, IFT-Nord, Kiel, GermanyTrial’s principal investigator: Reiner Hanewinkel, hanewinkel@ift-nord.de; scientific coordinator: Matthis Morgenstern, morgenstern@ift-nord.de.
Role of sponsor {5c}	The sponsor is non-commercial. The sponsor ensures quality management, qualified and trained personnel, study protocol compliance, and submission of relevant study documents to the ethics committee and regulatory authorities. He supports the study with trial unit facilities and study personnel.

## Introduction

### Background and rationale {6a}

Substance-related and addictive disorders typically have their onset in early adolescence and belong to the most common psychological disorders among emerging adults [1, 2]. In Germany, the group of 18- to 20-year-olds has the highest risk of addiction diagnoses regarding alcohol (6.4%), cannabis (1.5%), amphetamines (0.4%), and cocaine (0.3%) according to population-based survey data [3]. Research has consistently shown that the period of emerging adulthood [4] is not only associated with a readiness to engage in risky and unhealthy behavior such as substance abuse but also with limited capacities for self-regulation and (substance use-related) habit formation [5].

The proportion of young people affected by addictive behaviors increases if non-substance-related or “behavioral” addictions, particularly Internet-related disorders, are also taken into account [6]. Gambling disorder and (screen-related) gaming disorder seem to be similar to substance-related addictive disorders regarding their clinical appearance, etiology, comorbidity, and therapeutic responsiveness [7, 8]. Moreover, they are of increasing epidemiological relevance (even more so during the current COVID-19 pandemic conditions, see [9]) and have therefore recently been included in the revised classification of Substance-Related and Addictive Disorders in the 11th edition of the International Classification of Diseases (ICD-11) of the World Health Organization (WHO) (see [10]).

The school setting is particularly relevant for prevention efforts as it represents a central developmental context where a large number of individuals can be reached with comparatively little effort [11]. The majority of evaluated school-based preventive intervention programs apply only to regular schools, while there has been a lack of comparable programs for vocational schools [12]. However, in Germany, a substantial proportion of young people leaves the regular school system during adolescence and starts a vocational education, typically from the age of 16 [13, 14]. Vocational education in Germany largely takes place in the “dual system,” in which trainees are employed in a company and complete the practical part of their training there, while also attending vocational school, where the theoretical part of the training takes place.

Recent studies suggest that vocational students represent an important target group for prevention efforts and health promotion. In a survey including 5688 German vocational students, 40.5% of the participants reported a positive screening result for problematic alcohol use and 3.6% reported a level of cannabis use that puts them at serious risk for addiction [15]. The percentage of vocational students that reported daily smoking (41%) was 2.4 times greater than their age-matched peers in the general population. With regard to addictive behaviors such as online and offline gambling and digital media use, there are currently no data available that estimate the specific risks of vocational students. However, surveys indicate that media-related problems are particularly widespread among adolescents and young adults of typical vocational school age [6, 16].

A promising school-based prevention approach is a voluntary commitment to abstain from or reduce habitual behaviors like substance use. For example, one of the most widespread programs for the prevention of smoking in secondary schools in Germany is the smoke-free class competition “Be smart – don’t start.” At the core of the program is a joint voluntary commitment of the school class to not smoke for a period of 6 months. It focuses on influencing social norms, promoting self-regulation, and addressing social influences by deploying cognitive-behavioral intervention techniques [17]. Overall, the evidence based on process and rigorous outcome evaluations, long-term and iatrogenic effects, and cost-benefit efficiency are convincing, even in comparison with other programs (see [18]). An adaptation of this approach has also been shown to be effective in the prevention of binge drinking among older adolescents in the regular school setting in a randomized study [19]. Furthermore, the results of a recent controlled study in Germany [20] suggested that a 20-min reduction of daily social media use over a period of 2 weeks is positively associated with well-being and a healthier lifestyle among students. Specifically, the reduction in social media use not only reduced social media use intensity and the level of addictive symptoms but also the amount of daily smoked cigarettes over a period of 3 months.

### Objectives {7}

The present study aims to transfer the approach of voluntary abstinence to the vocational school setting and to evaluate the feasibility and effectiveness of this approach using a randomized design. It is expected that the intervention will increase awareness of habitual behaviors (e.g., substance and digital media use) and lead to a measurable reduction of these behaviors even after the end of the abstinence or reduction period.

### Trial design **{8}**

**Fig. 1:**
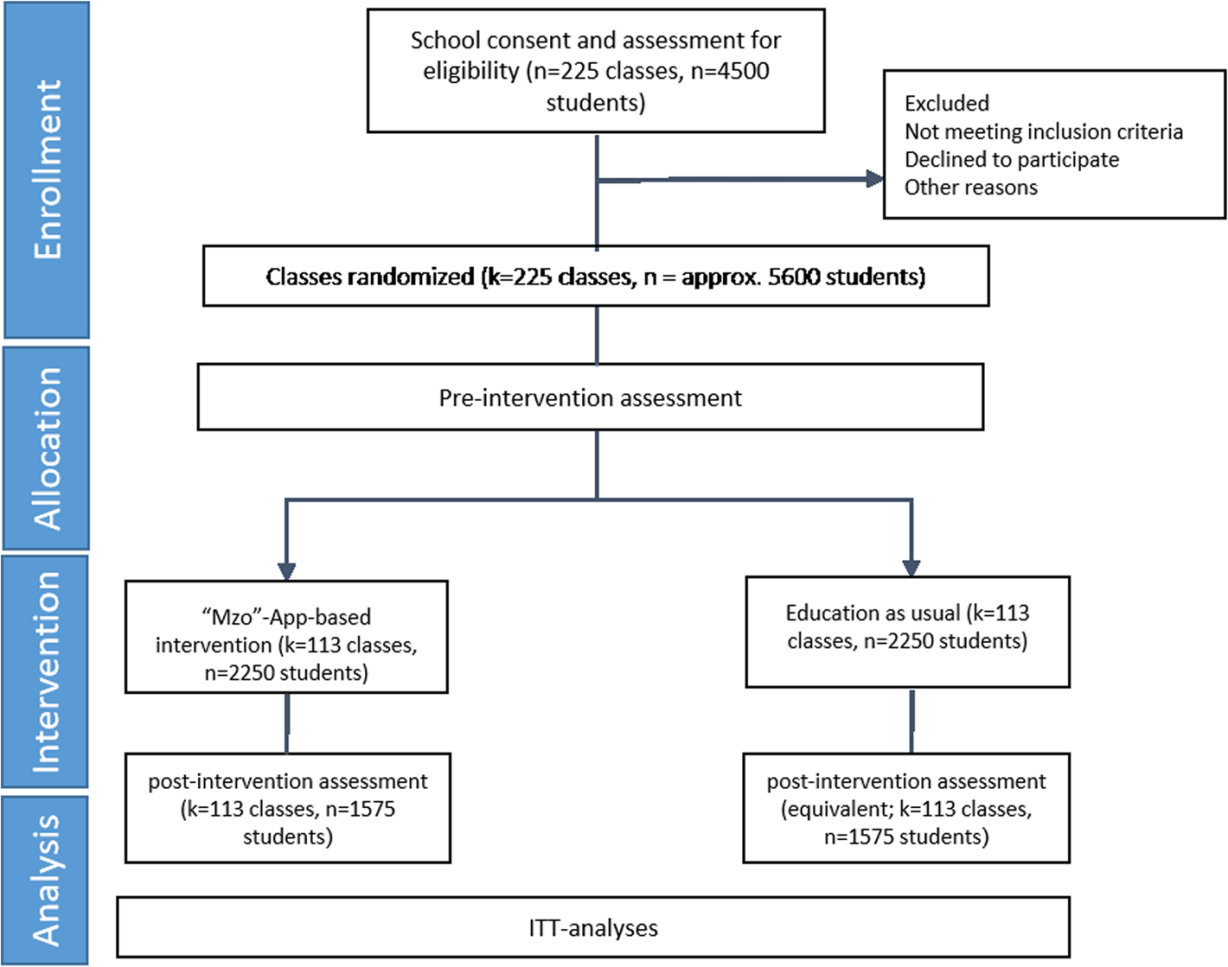
Consolidated Standards for Reporting Trials (CONSORT) diagram: study design and participant flow

The current study uses a cluster-randomized controlled exploratory trial with two arms, an intervention group (IG), and a waitlist control group (CG) with pre-post assessments. All study participants will be randomly assigned to one of the two groups on the class level (Fig. 1).

## Methods: participants, interventions, and outcomes

### Study setting {9}

The study will be conducted in vocational schools in the three German federal states of Schleswig-Holstein, Bavaria, and Hamburg. Schools were not systematically selected to represent the complexity of the vocational school system in Germany [14]; therefore, they vary in size, regional area, urbanicity (rural vs. urban), and vocational areas (e.g., business administration, industrial-technical professions, personal services).

### Eligibility criteria {10}

All vocational students will be eligible to participate in the study if they or one of their legal guardians (if the student is under the age of 16) provide written informed consent.

The age range of students attending vocational schools in Germany is broad. The mean age of students when starting vocational training is 19.7 years; approximately 11% of the students are aged 16 years or younger or 24 years or older [14].

### Who will take informed consent? {26a}

Trial participants and, if under the age of 16, their legal guardians will be provided with sufficient verbal and written information about the study’s purpose and procedures, information on confidentiality and data protection procedures, possible advantages and disadvantages of participation, and the option to withdraw from the study at any time and without any given reason. They are informed that participation in the study is completely voluntary. Written informed consent will be provided to and obtained from all participants prior to study enrollment by members of the study team.

### Additional consent provisions for collection and use of participant data and biological specimens {26b}

The consent includes the participant’s agreement to the collected data being used and published in an anonymized form for research purposes. This trial does not involve collecting biological specimens for storage.

### Interventions

#### Explanation for the choice of comparators {6b}

The comparator is an education-as-usual waitlist control group. All participants will be randomly assigned to either the app-based intervention group or the control group. The participants of the control group will continue routine activities during the (approximately) 6-week waiting period. This time frame was chosen because the intervention (use of the Mzo app) lasts 2 weeks and the primary trial outcomes concern past-month substance and media use. After post-assessment, participants of the control group will receive full access to the intervention app. Crossover and influence from peers in the experimental group can be considered low due to class-wise randomization and the nature of the vocational school setting (e.g., presence at school varies for each vocational area, and students are not present at school on a daily basis).

#### Intervention description {11a}

“Meine Zeit ohne” [*my time without*] (MZo) is an app-based intervention that aims to encourage users to voluntarily abstain from an individually relevant habitual behavior or to reduce it to a degree that is subjectively considered significant or a “challenge” for a period of 2 weeks. After downloading the app (downloadable for devices running on iOS 11.0 or higher/Android 6 or higher) and log in with a password, students can set their individual challenges. For the next 2 weeks, participants receive push notifications on a daily basis and are asked for feedback about whether they have reached their goal of the preceding day. MZo primarily targets consumption behavior (both substance-related and non-substance-related behavior, i.e., digital/screen-based media-related behavior such as gaming and/or social media use).

#### Criteria for discontinuing or modifying allocated interventions {11b}

Participants in the intervention group can discontinue the use of the intervention app at any time by deleting the app from their mobile devices. The intervention, i.e., the use of the app, does not require any contact with the study team.

#### Strategies to improve adherence to interventions {11c}

The implementation of the MZo challenge is entirely app-based. The effort required to use the app can be considered minimal. Adherence to the intervention is facilitated by an easy-access procedure (log-in and user authentication are only required once after the download of the app) and daily push notifications. The app provides the opportunity to choose individual behavior change goals from a broad spectrum including an undefined goal category which can be specified by the individual user.

#### Relevant concomitant care permitted or prohibited during the trial {11d}

Participation in the current trial has no impact on possible concomitant care during the time of the trial.

#### Provisions for post-trial care {30}

All participating schools are provided with contact information of the study sites and can access evidence-based information material on substance use and substance use prevention via the project website.

### Outcomes {12}

#### Primary outcomes

**Fig. 2:**
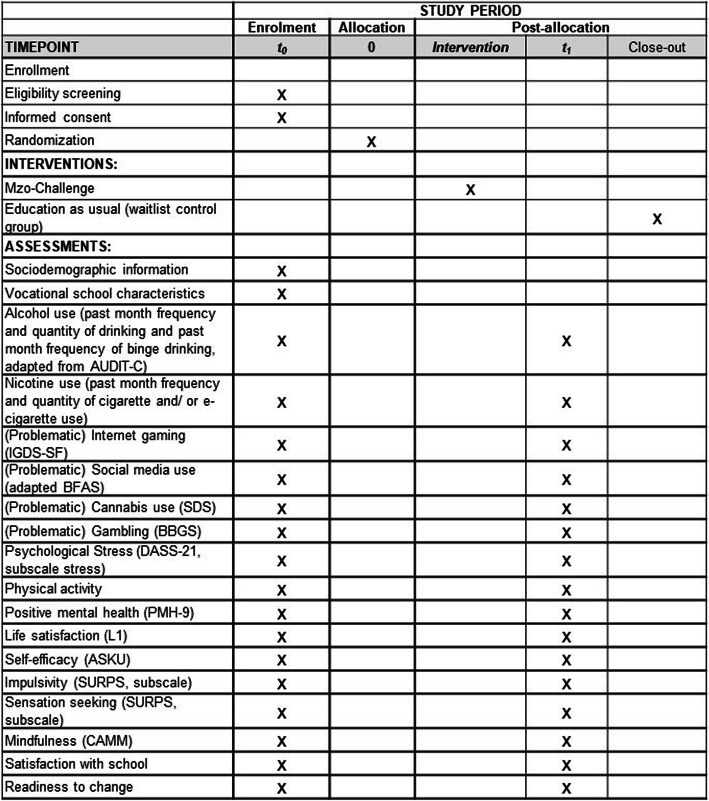
Standard Protocol Items: Recommendations for Interventional Trials (SPIRIT) diagram detailing the trial activities and measures and their timing

Outcome measures at each time point are shown in Fig. 2. One of the primary outcomes is self-reported use of alcohol, nicotine (cigarettes and/or electronic cigarettes), and digital media (gaming and social media) in the past month. Measures for alcohol use are based on [21] and include frequency of drinking (1 = “never” to 5 = “four times a week or more”), quantity of alcoholic drinks on a typical drinking day (1 = “1 or 2 alcoholic drinks” to 5 = “10 or more alcoholic drinks”), and frequency of binge drinking (more than 6 alcoholic drinks on one occasion; 0 = “never” to 4 = “daily or almost daily”). Nicotine use is assessed based on students’ reports on the number of days using cigarettes and/or electronic cigarettes and their quantity per day. Problematic Internet gaming is assessed using the Internet Gaming Disorder Scale-Short-Form (IGDS-SF [22];), German version ([23]; 9-items: e.g., “Have you continued your gaming activity despite knowing it was causing problems between you and other people?”; 1 = “never” to 5 = “very often”). Problematic social media use was assessed using an adapted version of the brief version of the Bergen Facebook Addiction Scale (BFAS [24];; German version: [25]). The scale consists of 6 items (e.g., “Felt an urge to use social media more and more”; 1 = “very rarely” to 5 = “very often”). All primary outcome measures (in their German versions) have been used in previous studies and can be considered psychometrically valid.

#### Secondary outcomes

A number of individual-level secondary outcome measures were selected based on their health-related relevance and associability with the conceptual intervention approach. These outcomes are as follows:

- Problematic cannabis use (Severity of Dependence Scale (SDS), 5 items [26]; German version: [27])
- Problematic gambling (Brief Biosocial Gambling Screen (BBGS), 3 items [28])
- Psychological stress (Depression Anxiety Stress Scales 21 (DASS-21), subscale stress, 7 items [29]; German version: [30])
- Impulsivity and sensation seeking (Substance Use Risk Profile Scale (SURPS), subscales impulsivity and sensation seeking, 2 items each [31])
- Mindfulness skills (Child and Adolescent Mindfulness Measure (CAMM), 10 items [32])
- Physical activity (past month frequency of physical activity/sports, single item, self-constructed)
- Positive mental health (Positive Mental Health Scale (PMH), 9 items [33])
- Life satisfaction (L1, General Life Satisfaction Short Scale, single item [34], German version [35])
- General self-efficacy (Short Scale for Measuring General Self-efficacy Beliefs (ASKU), 3 items [36])
- Readiness and confidence to quit or reduce the use of alcohol, nicotine, cannabis, gaming, social media, and gambling (readiness/confidence ruler (10 = “not at all ready/difficult to change” to 100 = “very ready/difficult to change”) assessing readiness and confidence in quitting or reducing each behavioral outcome; based on [37])

For a list of included secondary outcomes, see Fig. 2. Additionally, we assess socio-demographic data (age, gender, migration background, socio-economic status), progress of vocational education, educational sector, and frequency of in-school education.

#### Participant timeline {13}

In Fig. 1, trial procedures from enrollment to the end of the trial are illustrated.

#### Sample size {14}

The sample size is calculated to detect a minimum relative difference of 20% between both groups at post-assessment. This effect was based on previous studies on the effectiveness of substance use prevention programs [11, 19] and takes a clustered data structure and an intra-class correlation (ICC) of 0.032 as well as a drop-out rate of 30% on the student level into account (based on [15]). The required sample size for 80% power to detect between-group differences at the 0.05 level is 4500 students (2250 students per condition) from 225 classes.

#### Recruitment {15}

The three study sites cooperate with local school supervisory authorities, which support the project and allow the recruitment of a convenient sample of vocational schools. Class teachers and other school staff (such as school social workers) at those schools are informed about the study in teacher conferences. This information includes access to the project website which features explanation videos about the MZo app and the implementation of the study. Teachers will be provided a login code to test the MZo app prior to implementation in their class. If teachers decide to participate in the study, two appointments for data collection and for the introduction of the intervention will be scheduled. Consent to participate in the study on the student level has to be provided prior to baseline assessment.

### Assignment of interventions: allocation

#### Sequence generation {16a}

Randomization will be performed at the class level. The randomization sequence has been created [38] using the program “Randomization in Treatment Arms” (https://www.evidat.com/rita). Classes of vocational schools will be stratified by class characteristics and paired into dyads of similar classes. The first member of a dyad will have a 50% chance to be assigned to the intervention group, and the remaining class will automatically be assigned to the other trial arm.

#### Concealment mechanism {16b}

In the temporal order of study inclusion, allocation of classes will be made according to the randomization plan to either the MZo intervention or the control group. Participants, teachers, and schools will have no influence on the allocation process and assignment of classes to either group, but they will not be blind to randomization.

#### Implementation {16c}

Random allocation of classes within schools to either the MZo intervention or the control group using the randomization plan takes place at each study site under the responsibility of the local study team. All students who give their written consent (electronically) to participate in the study will be enrolled. Unique identifiers are generated using an ID-generator software and will provide access to the electronic assessment portal. The flow diagram of the study design is depicted in Fig. 1.

### Assignment of interventions: blinding

#### Who will be blinded {17a}

All participants, teachers, and schools will be blinded to the randomization and allocation process. However, they will not be blind to the MZo intervention, as participants of the control group will be informed that they will receive access to the MZo app after post-assessment. Data analysts will be blinded.

#### Procedure for unblinding if needed {17b}

The design is open-label with only data analysts being blinded so unblinding will not occur.

### Data collection and management

#### Plans for assessment and collection of outcomes {18a}

Data will be collected class-wise in schools. The baseline assessments will take place directly before the intervention and the post-assessments at least 30 days after the end of the intervention. All measures are assessed via the online system “SoSci Survey,” program version 3.2.12 (15.02.2021). All collected data is entered using participants’ personal smartphones. If individual smartphones are unavailable or not functioning due to technical problems, the study teams will provide tablets to ensure data collection. To guarantee a smooth online data assessment, the study teams will also provide a portable LTE router for a stable Internet connection in the classroom if necessary. As for the MZo app and the online assessment portal, compatibility with current Android and iOS devices is given.

#### Plans to promote participant retention and complete follow-up {18b}

According to their allocation, study participants will complete the online questionnaires in class. Unique identifiers are distributed by the study team and can be used to access the questionnaire for pre- and post-assessments. The same identifier can also be used to log in to the MZo app. To maintain high retention rates between pre- and post-assessments, participants are encouraged to take a smartphone picture of their unique identifier. To maximize data completeness, vocational students who are absent from school on the day(s) of the assessment will be contacted in a coordinated approach by their teachers.

#### Data management {19}

Online assessment data will automatically be transferred to a local server at the study site in Kiel (SH), minimizing errors of data entry. All participant data is handled in accordance with the General Data Protection Regulation (GDPR). All data will be maintained confidentially before, during, and after the trial and is stored securely at the study site in Kiel with access only by dedicated study team members.

#### Confidentiality {27}

All data is collected using 6-digit unique identifiers to access the online questionnaires. The first two digits of this code identify the specific school, the next two digits identify a particular class, and the last two digits that identify an individual participant are randomly distributed. Single individuals cannot be identified.

#### Plans for collection, laboratory evaluation, and storage of biological specimens for genetic or molecular analysis in this trial/future use {33}

No biological specimens will be collected.

## Statistical methods

### Statistical methods for primary and secondary outcomes {20a}

Analyses will account for the clustering of individuals in school classes and will be reported following CONSORT standards. Trial variables will be analyzed by the intervention arm, into which participants were randomly assigned. Continuous outcomes will be reported for each trial arm using means and standard deviations, and binary outcomes will be reported for each trial arm using numbers and percentages. The main intervention effects will be tested by means of logistic or linear multi-level/random effects regression models with the levels “classes” and “individuals,” whereby group and time variables as well as the interaction term group × time are used. Primary between-group analyses will be adjusted for baseline scores of the outcome variable, relevant covariates (baseline prognostic factors that are theoretically associated with outcomes, including, but not limited to age, gender, and migration background as well as the personality traits sensation seeking and impulsiveness), and those variables used to stratify randomization.

### Interim analyses {21b}

No interim analysis will be performed.

### Methods for additional analyses (e.g., subgroup analyses) {20b}

For sensitivity analysis, unadjusted and complete case (without imputation of missing data) analyses will additionally be carried out for primary and secondary outcomes. Potential moderators, such as individual differences in socio-demographic factors (age, sex, etc.) or vocational school-level factors (i.e., vocational sector, etc.), and potential mediators (mechanisms of action) such as self-efficacy or abstinence/reduction-related control beliefs will be explored in interaction analyses.

### Methods in analysis to handle protocol non-adherence and any statistical methods to handle missing data {20c}

Primary analyses will be based on the intention-to-treat population, thus including data from all participants who provide baseline data within a school class that was previously randomly assigned to one of the two trial conditions. Multiple imputation methods will be used to estimate missing data.

### Plans to give access to the full protocol, participant-level data and statistical code {31c}

Anonymized study data and statistical codes will be made available upon request given that data protection according to GDPR and ethics according to ethical approval are ensured.

### Oversight and monitoring

#### Composition of the coordinating center and trial steering committee {5d}

The coordinating center is the primary sponsor of this trial and is responsible for study supervision. The coordinating investigators at the three study sites form a steering committee for the study. They meet regularly and are responsible for the critical review of the study design, the study protocol, the data management, and all the study-related documents. The coordinating investigators provide oversight on the trial and support the study team members who conduct and provide day-to-day organizational support for the trial at each site. Furthermore, the coordinating investigators are responsible for the trial registration, revisions of the study protocol and application/amendments to the ethics committee, and scheduling of regular team meetings. All involved investigators and team members ensure compliance with the study protocol.

#### Composition of the data monitoring committee, its role, and reporting structure {21a}

A Data Monitoring Committee is not considered necessary as this is a low-risk intervention.

#### Adverse event reporting and harms {22}

Information on the occurrence of adverse events will be documented. Adverse events related to the assessment or use of the MZo app during the 2-week intervention period will be recorded by the coordinating investigator.

#### Frequency and plans for auditing trial conduct {23}

The Project Group meets at least bi-weekly to review the trial conduct throughout the trial period. There will be no external auditing during the trial.

#### Plans for communicating important protocol amendments to relevant parties (e.g., trial participants, ethical committees) {25}

All protocol deviations or modifications will be documented; all substantial protocol amendments will be communicated to the ethics committee of the Center for Psychosocial Medicine at the University Medical Center Hamburg-Eppendorf, the responsible school authorities at each study site (Center for Education Monitoring and Quality Development at schools in Hamburg, IfBQ; the Center for Prevention at the Institute for Quality Development at Schools in Schleswig-Holstein, IQ.SH; and the Bavarian State Ministry for Education and Cultural Affairs), and the German Clinical Trials Register, DRKS (DRKS00023788).

#### Dissemination plans {31a}

The study results will be presented at conferences and symposia and will be submitted for publication in relevant journals.

## Discussion

The present study aims to transfer the voluntary abstinence approach that has been established in regular schools to the vocational school setting and extends it to digital media-related addictive behaviors. This will be the first time that a prevention program for vocational schools will be developed and rigorously evaluated in a randomized study in Germany. Moreover, the scientific evaluation of a fully app-based intervention of this approach extends the literature on prevention measures based on the voluntary commitment which up to now has been mainly established in the form of the class competition “Be smart – don’t start” [17].

If the trial results suggest that the approach is feasible and effective, this could provide a much needed evidence-based, relatively low-cost, and scalable program to reduce substance use and Internet-related problems at vocational schools in Germany. All MZo materials (explanation video, app, MZo website) is designed to require minimal effort and resources for schools/teachers. The “core intervention material” is the MZo app which can be considered credible for the target group since vocational students participated in the process of conceptual development to pilot testing for usability and acceptability.

To facilitate recruitment of vocational schools, the cluster RCT design is limited to a convenience sample of vocational schools willing to participate and a pre-post design. While this approach may result in selection bias and limit conclusions regarding long-term effectiveness, we consider this approach acceptable given that the study evaluates the effectiveness of a newly developed intervention. Although the recruited sample cannot be considered representative of vocational schools in Germany, the generalizability of the results is facilitated by the inclusion of a broad range of vocational sectors (i.e., service industries, business and administrative professions as well as industrial-technical professions) within schools from three different states across Germany. Moreover, implications for practice will be gained. The study will contribute to the knowledge on how to effectively implement the MZo intervention in (vocational) schools. Finally, the inclusion of a range of potentially relevant secondary outcomes, covariates, moderators, and mediators in a reasonably large sample is expected to add to a differential understanding of the trial results.

## Trial status

Ongoing trial. Recruitment of participants started in March 2021 and is expected to be completed in April 2022. Protocol version #1; protocol version date 1 April, 2022.

## Abbreviations

ASKU: Short Scale for Measuring General Self-efficacy Beliefs
AUDIT – C: Alcohol Use Disorders Identification Test
BBGS: Brief Biosocial Gambling Screen
BFAS: Bergen Facebook Addiction Scale
BMG: Federal Ministry of Health Germany
CAMM: Child and Adolescent Mindfulness Measure
CG: Control condition (group)
CONSORT: Consolidated Standards for Reporting Trials
DASS-21: Depression Anxiety Stress Scales 21
DRKS: German Clinical Trials Register
GDPR: General Data Protection Regulation
ICC: Intra-class correlation
ICD-11: International Classification of Diseases
IfBQ: Center for Education Monitoring and Quality Development at schools in Hamburg
IG: Intervention group
IGDS-SF: Internet Gaming Disorder Scale-Short-Form
IQ.SH: Center for Prevention at the Institute for Quality Development at Schools in Schleswig-Holstein
L1: General Life Satisfaction Short Scale
MZo: “Meine Zeit ohne” [my time without]
PMH: Positive Mental Health Scale
RCT: Randomized controlled trial
SDS: Severity of Dependence Scale
SPIRIT: Standard Protocol Items: Recommendations for Interventional Trials
SURPS: Substance Use Risk Profile Scale
WHO: World Health Organization
